# NORG - Nitrogen ORGanizer: a novel web-based system for cryopreservation and freezer management

**DOI:** 10.1093/database/baag037

**Published:** 2026-07-11

**Authors:** Marvin Keller, Maximilian Przybilla, Jannik Sehring, Kai Schmid, Thomas Kauer, Felix Dörmer, Nadja Ritschel, Annika May, Hannah Schlierbach, Gudrun Schmidt, Kerstin Leib, Chiana Stephan, Elena Jenike, Carmen Selignow, Angelina Frank, Vincent Umathum, Anne Schänzer, Attila Németh, Keywan Sohrabi, Till Acker, Nick Heller, Hildegard Dohmen, Daniel Amsel

**Affiliations:** Institute of Neuropathology, Justus-Liebig-University Giessen, Arndtstr. 16, 35392 Giessen, Germany; Institute of Neuropathology, Justus-Liebig-University Giessen, Arndtstr. 16, 35392 Giessen, Germany; Institute of Neuropathology, Justus-Liebig-University Giessen, Arndtstr. 16, 35392 Giessen, Germany; Institute of Neuropathology, Justus-Liebig-University Giessen, Arndtstr. 16, 35392 Giessen, Germany; Institute of Neuropathology, Justus-Liebig-University Giessen, Arndtstr. 16, 35392 Giessen, Germany; Institute of Neuropathology, Justus-Liebig-University Giessen, Arndtstr. 16, 35392 Giessen, Germany; Institute of Neuropathology, Justus-Liebig-University Giessen, Arndtstr. 16, 35392 Giessen, Germany; Institute of Neuropathology, University Hospital Giessen and Marburg, Arndtstr. 16, 35392 Giessen, Germany; Institute of Neuropathology, University Hospital Giessen and Marburg, Arndtstr. 16, 35392 Giessen, Germany; Institute of Neuropathology, University Hospital Giessen and Marburg, Arndtstr. 16, 35392 Giessen, Germany; Institute of Neuropathology, University Hospital Giessen and Marburg, Arndtstr. 16, 35392 Giessen, Germany; Institute of Neuropathology, University Hospital Giessen and Marburg, Arndtstr. 16, 35392 Giessen, Germany; Institute of Neuropathology, University Hospital Giessen and Marburg, Arndtstr. 16, 35392 Giessen, Germany; Institute of Neuropathology, University Hospital Giessen and Marburg, Arndtstr. 16, 35392 Giessen, Germany; Institute of Neuropathology, Justus-Liebig-University Giessen, Arndtstr. 16, 35392 Giessen, Germany; Institute of Neuropathology, Justus-Liebig-University Giessen, Arndtstr. 16, 35392 Giessen, Germany; Institute of Neuropathology, Justus-Liebig-University Giessen, Arndtstr. 16, 35392 Giessen, Germany; Institute of Neuropathology, Justus-Liebig-University Giessen, Arndtstr. 16, 35392 Giessen, Germany; Faculty of Health Sciences, University of Applied Sciences Mittelhessen, Ostanlage 45, 35390 Giessen, Germany; Institute of Neuropathology, Justus-Liebig-University Giessen, Arndtstr. 16, 35392 Giessen, Germany; Faculty of Health Sciences, University of Applied Sciences Mittelhessen, Ostanlage 45, 35390 Giessen, Germany; Institute of Neuropathology, University Hospital Giessen and Marburg, Arndtstr. 16, 35392 Giessen, Germany; Institute of Neuropathology, Justus-Liebig-University Giessen, Arndtstr. 16, 35392 Giessen, Germany

## Abstract

The preservation of samples, whether they are from humans, cell lines, animals, plants, or other resources, usually follows a similar process. They are packaged in appropriate containers, whether tubes for cryogenic containers or boxes in freezers or other systems. For this biological sample database to be and remain useful, the samples must be appropriately cataloged and consecutively maintained in a document. After all, only when it is clear where which sample is located, the biological database has a value. In many cases this documentation is done in local spreadsheets and saved in e.g. CSV or XLSX format. In some cases, there are even handwritten records to comprehend where which sample is located. The intention behind a biological database is mainly the collection of rare material, but also the withdrawal of this material from the storage for further analysis, partly in-house but presumably also with external cooperation partners. If a sample is now taken from a container that has already been completely filled and stored, this empty space can only be re-allocated with a sample with great administrative effort, since new samples would otherwise be stored in the container that is currently active. With NORG we address this problem. NORG offers a stand-alone software solution via webservices to catalog e.g. nitrogen tanks and store the contents digitally with a color-coding for the filling level of the storages. In addition, NORG can trace who sent which sample to whom and when, for example to have it analyzed by a cooperation partner.

**Database URL:**  https://gin-norg-dev.med.uni-giessen.de/

## Introduction

The systematic long-term preservation of biological samples is common practice in many laboratories. For several decades, especially cryopreservation, which is the freezing and storage of samples at low temperatures, e. g., -196°C in liquid nitrogen, has proven successful in preserving cells, tissues, and even organs [1–6]. To maintain these low temperatures and avoid thawing of samples, opening of the storage containers, such as nitrogen tanks, should be restricted as far as possible. Thus, the location and additional information of a respective sample is documented on an external medium, e. g., a book, which is commonly located directly next to the tank, or a digital spreadsheet. Both media types have the disadvantage that access is usually very limited and less flexible, as either physical or digital access to the list is required. In case of a paper-based list, like a book or printed spreadsheet, a typical workflow includes going to the corresponding room, searching for the sample in the list, crossing it out or entering a new sample with respective additional information. In the case of a digital list, the current version of the list must be available to be updated. The first variant becomes cumbersome as soon as the number of samples increases or changes are frequent. The latter method is more comfortable but has several other disadvantages. First, file duplications with different versions might arise when sharing the spreadsheet with co-workers. Furthermore, race conditions could occur if more than one person is currently working on a centrally deposited file. Sometimes, it is also desired to grant specific rights to a certain group of people. Obviously, users who are actively archiving samples need write permission, whereas a read permission might be sufficient for users who only search for samples and their associated information. Another problem posed by the previously described systems is the possibility of misspellings and transposed numbers. Especially handwriting can be challenging and sometimes misleading, exemplified by an entire research field tackling this issue for decades [7]. Managing multiple storage containers can also lead to increased effort, for example, if the capacity differs from one system to another. Disposing of decommissioned storage can also be problematic if, for example, samples are overlooked in books or spreadsheets.

In our laboratory, we have encountered precisely these problems. To address these issues, we developed NORG (Nitrogen ORGanizer), a novel web-based system for cryopreservation and freezer management, which was designed in close collaboration with our laboratory technicians to meet their needs. It can be installed on a local server and is accessed via common web browsers. It enables assigning specific roles to users to manage read and write permissions. Users with read permission can see which samples are available or were recently sent by whom, e. g., to collaboration partners. With NORG, misspellings of identifiers can be avoided when a barcoding system is used during the creation of samples. Moreover, the data is stored digitally and can therefore be read easily by humans as well as algorithms. Furthermore, NORG efficiently handles vacant spaces in tanks and refrigerators by visually color-coding slots for samples. In the NORG system, users with appropriate rights can create new patterns for storage systems and define how many spaces are available. If a new storage unit is purchased, it can be installed directly using the profile created previously. With NORG, decommissioned storage can easily be removed, if and only if it is empty according to the NORG system. In case a sample has been missed, the NORG system intervenes and refuses to delete the system. Furthermore, NORG can be adapted for usage with other storage systems besides nitrogen tanks, e. g., for freezers. To our knowledge, NORG is the first open-source system to provide a solution to these problems. We found another database that specializes in the management of laboratory animals [8], but this system did not fit our defined needs for a web-based cryopreservation and freezer management database, highlighting the need for NORG.

## Administration

### Installation

In designing NORG, we looked for good transferability to other research sites and institutes as well as ease of setup. We therefore decided to run the MariaDB database and the Laravel framework [9] in Docker [10] containers that are orchestrated via Docker-compose. The versions of the MariaDB and Laravel containers can be changed individually via the docker-compose file, although it is recommended to use the pre-defined versions as they were tested.

The NORG project is deposited under *https://github.com/Neuropathology-Giessen/GIN-NORG/* and can be cloned from there to a local server system that runs Linux. The NORG system is then started via ‘*docker-compose up*’ and after a short boot time, the administrator needs to execute a few commands inside the Laravel container by using ‘*docker exec norg_laravel*’. This should properly setup the NORG system, so that the administrator may log in via *‘admin@norg.de’* as username and ‘*adminpass*’ as password. It is highly recommended to change the user credentials after the first login. Further details for the installation can be found on the GitHub page.

### User administration and roles

New users may register on the login page themselves, but they need to be verified manually by the administrator, who assigns the novel user a predefined role. NORG comes by default with the roles ‘physician’, ‘office’, ‘lab technician’ and ‘administrator’. Initially the newly registered user has the ‘default’ role which comes without any rights. This is intentionally designed so that the administrator needs to modify the rights of the new user. Depending on the role they may interact differently with NORG. The physician and office users only have reading rights, as it is intended for this group of people to only check for available material or provide information about samples that are available or have been sent away. The users with the second most rights in NORG are the lab technicians. Here the idea was that in the laboratory the samples are prepared and collected. They are also archived in the laboratory, both physically and digitally. Of course, a user with administrator role has the rights to accept users and change their role. NORG deliberately avoids extensive role and rights management in order to keep the system lean and with less administrative effort.

### Backup and data recovery

To account for data security, we implemented a solution for automated backups of the entire database. Therefore, a crontab job can be established on the local server system. The execution of ‘crontab -e Command: * 0 * * * docker exec -i norg_mariadb mysqldump -u root bitnami_myapp >/path/to/GIN-NORG/public/sqldumps/NorgDBdump$(date +%Y%m%d).sql’ will set up a backup routine every midnight.

Of course, this command can be adapted. Here we assumed that the user and the password are still the default values. We also assumed that NORG is located at ‘*/opt*’, where we then save the database dump into the ‘*public/sqldumps/*’ folder. The naming of the backup takes the local server time to keep the database dumps distinguishable. It could also be beneficial to save the database dumps elsewhere, e.g. on a network storage. In case a data recovery is needed, the particular SQL file needs to be stored in the GIN-NORG folder, for example under ‘*public/sqldumps/*’, so that the default docker-compose may have access to the file.

## Database architecture

**Figure 1:**
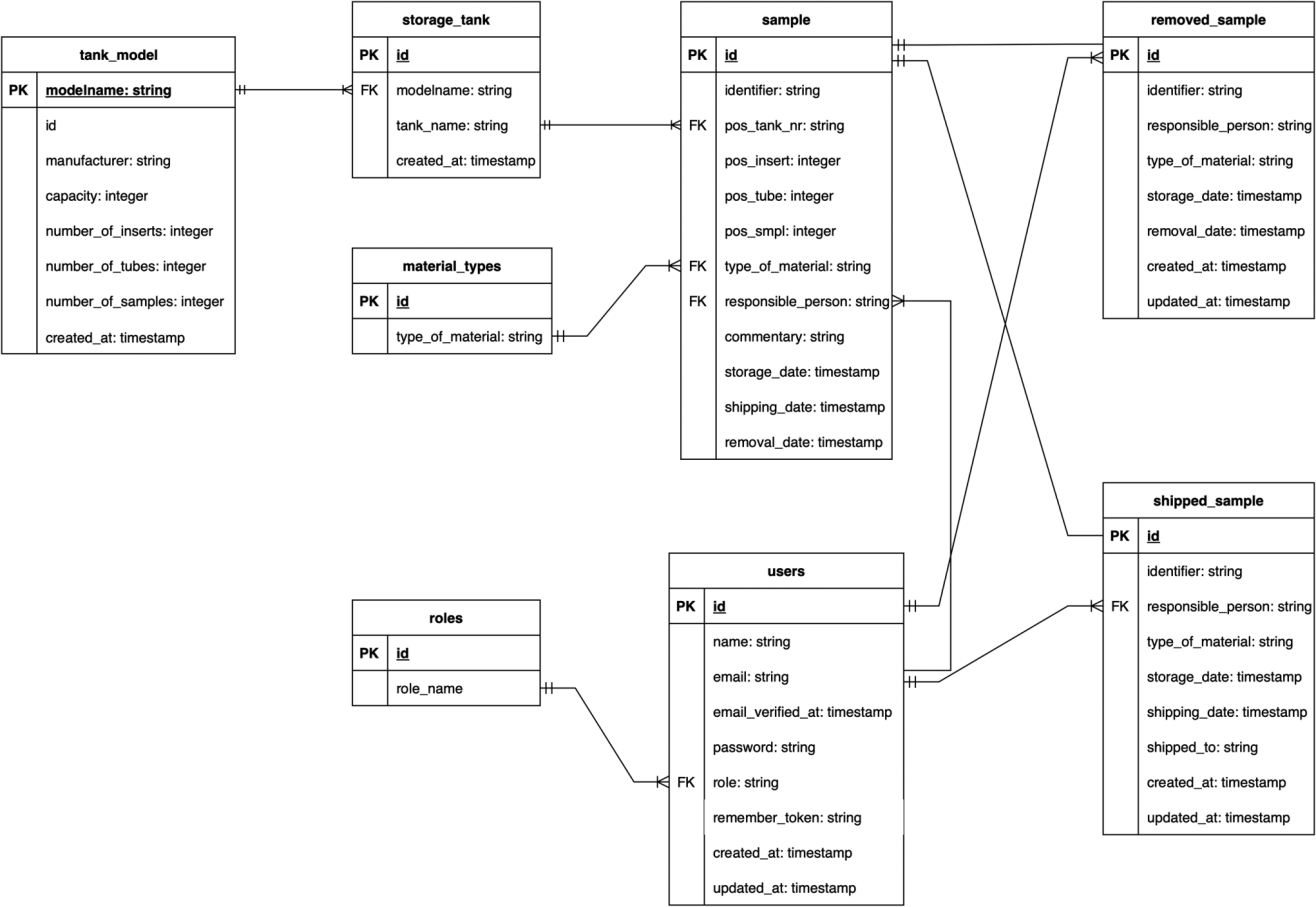
Database architecture of NORG: The sample Table contains the essential information of the stored sample. The ‘storage tank’ Table and its adjacent Table ‘tank model’ describe the storage that can be a nitrogen tank or anything similar. Users may have roles and can store, send and remove samples, but can also create or remove storage tanks. If a sample is shipped, the database saves the information of the sample and details about the shipment.

NORG is written in a relational database management system (RDBMS), namely MariaDB. The architecture is focusing on the sample and material types, but also has Tables for different storage systems, like tanks or freezers and a user management system (Fig. 1).

## Web interface and use cases

The NORG system is intended to be used as webservice. Therefore, it can be accessed via a standard web browser that has a connection to the local NORG server. By default, the web service is running on port 8000. This can be changed by the administrator, using the docker-compose file. Additionally, it is highly recommendable to secure the webservice via an encryption method by using NGINX [11] for example.

### View samples

**Figure 2:**
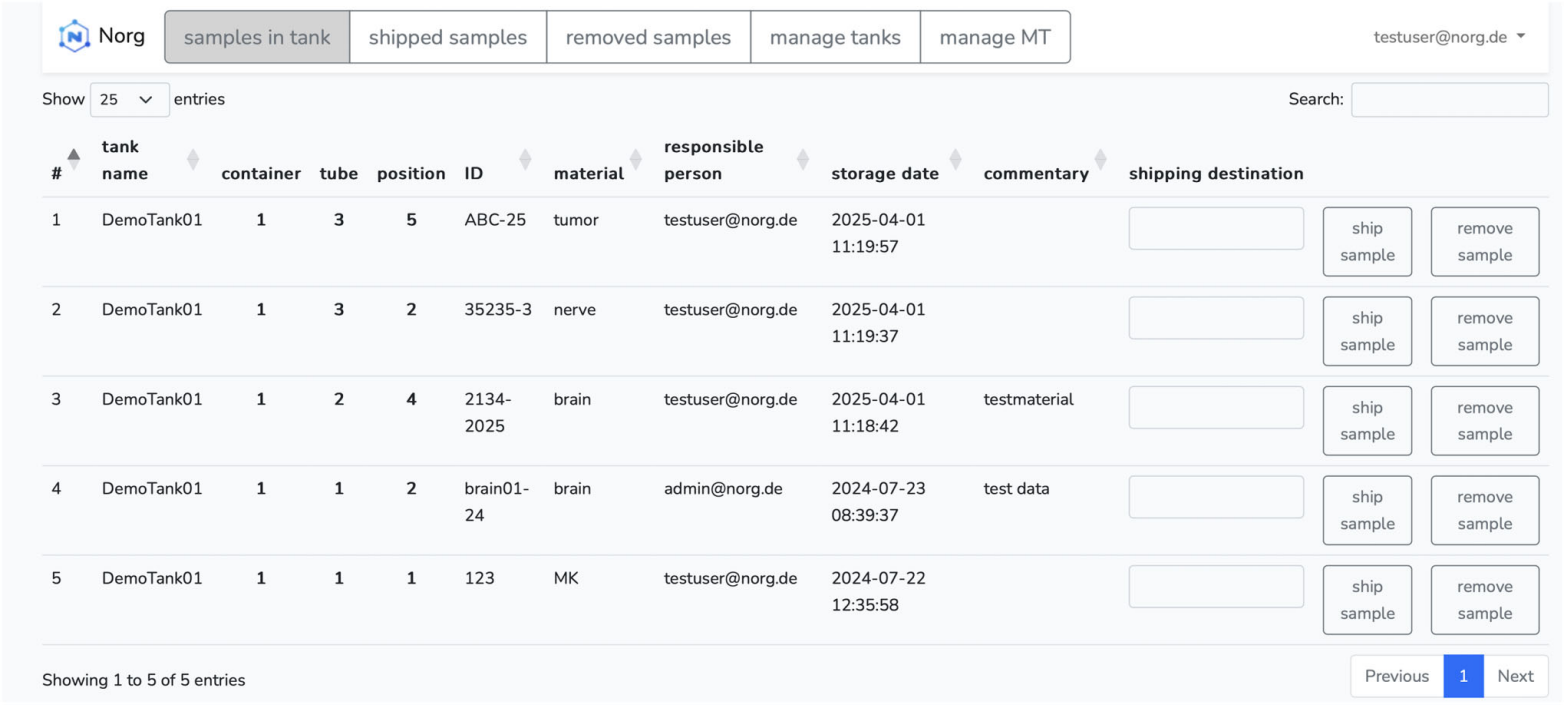
**Screenshot of NORG:** Viewing the samples in the database as a list. From here it is possible to ship samples or remove them permanently from the database. One may also see the exact location of the sample, as well as the person who was responsible for the sample and when the sample was stored.

One of the main purposes of NORG is to find samples in the database and their corresponding location in the storage (Fig. 2). This mode is available for all roles of NORG users. By clicking the ‘*samples in tank*’ tab, the NORG systems lists all archived samples in a Table. It is possible to search for a specific entry via ‘*ctrl + f*’ or via the search bar. The sample list displays the name of the sample, as well as its detailed location, together with the material, the responsible person, the storage date, free text comments and in case it has been send away, the shipping location. Outgoing from this list, one can ship the sample or remove it from the storage.

### Add samples

**Figure 3:**
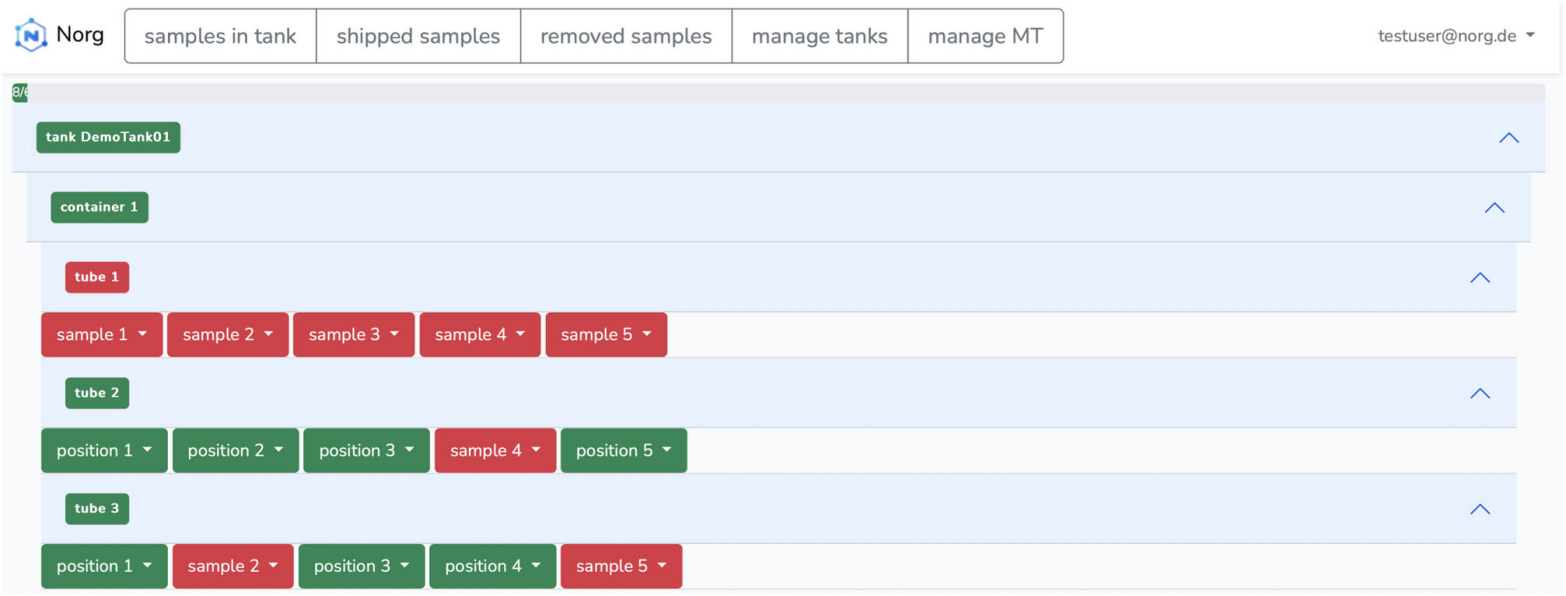
**Screenshot of NORG:** Selecting a free position in the storage system is very convenient in NORG. Red locations are fully occupied. A progress bar at the top of the container shows the overall filling.

The storages are colorized depending on the percentage of their filling (Fig. 3). In case they are green, more than 80% is free. If they turn yellow, they are filled between 80 and 99% and if they turn red, they are 100% filled. In this tab it is also possible to mark samples to be sent. After marking, they are automatically removed. They can then be found in the next tab, which displays all the samples that have been sent.

**Figure 4:**
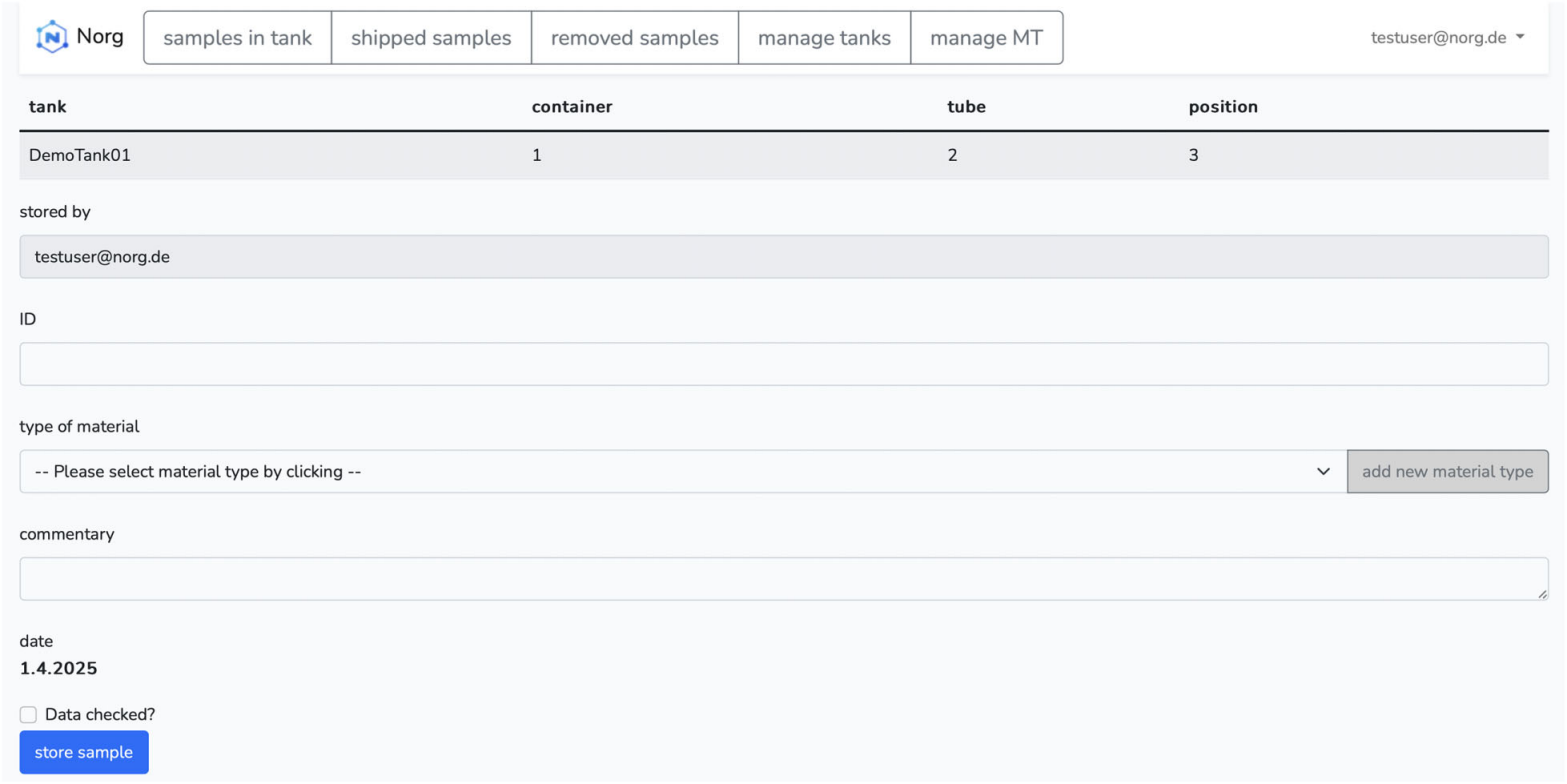
**Screenshot of NORG:** When adding a sample to the database, the cursor is directly in the ID field, enabling the user to scan a barcode with the ID. The other information must be selected manually. A checkbox at the end needs to be confirmed to ensure all information are correct.

For a quick and convenient data acquisition, NORG is designed in a way that it jumps with the cursor directly to the sample identifier field (Fig. 4). This enables the users to scan a sample via barcode and have the identifier directly available in the correct field. The type of the stored material can be chosen via a drop-down menu. The sample types can be customized using the ‘manage MT’ section. Before submitting a novel sample, the user is asked if every entry is correct via a checkbox.

### Send samples

**Figure 5:**
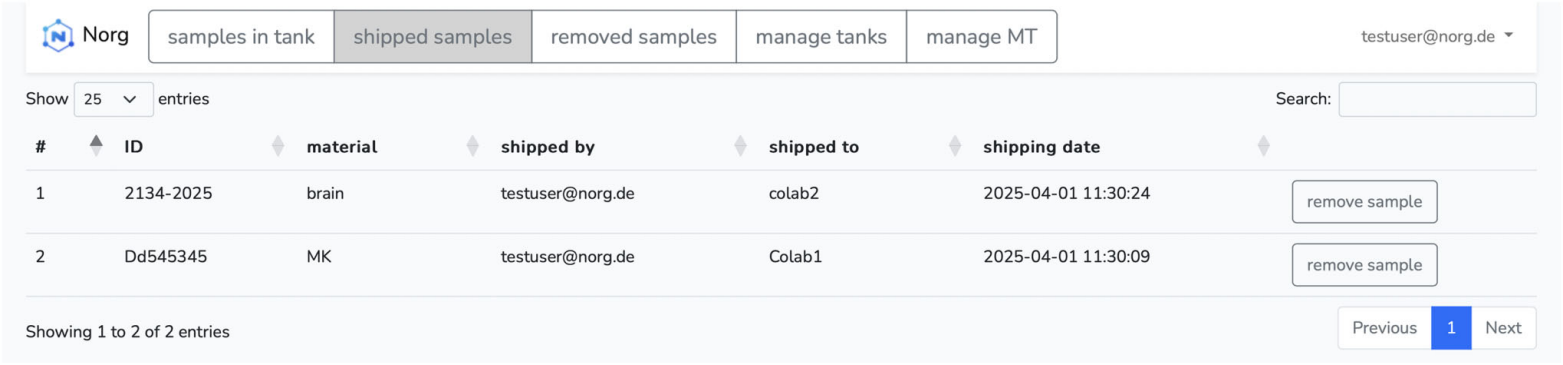
**Screenshot of NORG:** This menu lists all samples that have been send away and are currently not available in the local storage. One can conveniently remove them if they will not be sent back.

**Figure 6:**
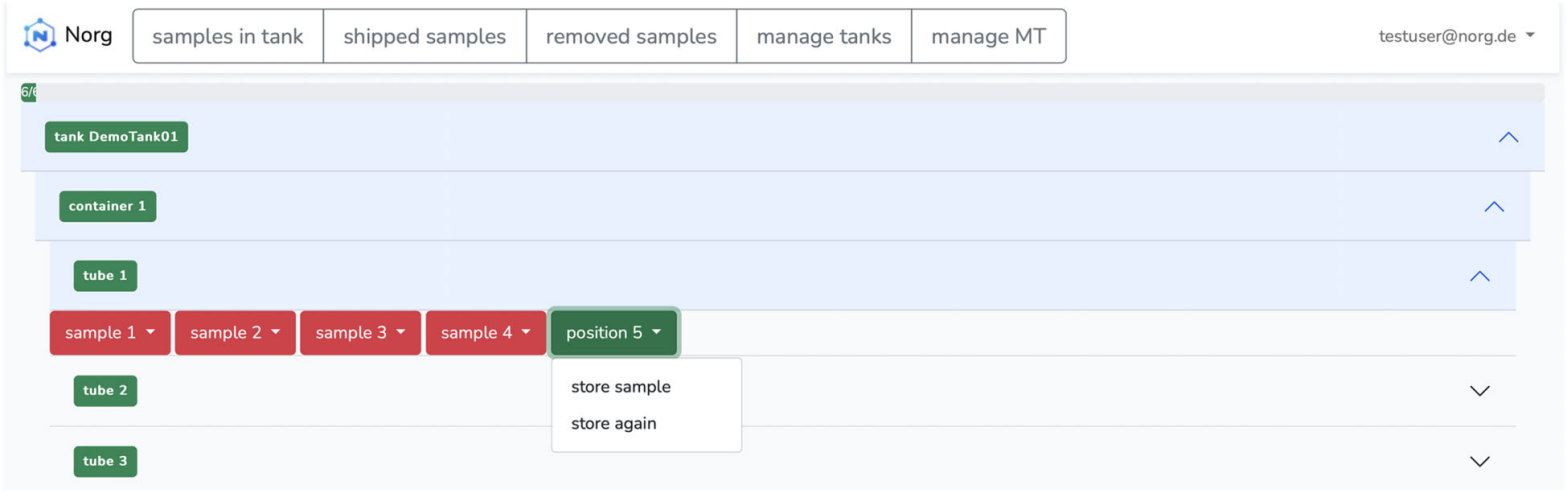
**Screenshot of NORG:** Selecting a free position in the storage system and restore samples back on place.

This tab contains a list of all samples that have been sent (Fig. 5). When a sample is returned, it can also be archived again from the ‘*Norg’* home tab with the ‘*store again*’ button on chosen position in container (Fig. 6). It is possible to select a new place in the container, as it may happen that the previous place has been occupied by another sample in the meantime. Primarily you can see here the identifier, the material type, as well as the person who sent the sample, when and where. If the sample will not return, it can also be removed from the list (Fig. 5).

The NORG system allows users to efficiently manage shipped samples, facilitating their return to storage either in their original positions or in new locations within the tanks. Through the interface displayed, users can select specific samples from the list and utilize the ‘store sample’ button to execute the storage process. This functionality ensures that samples are accurately tracked and managed throughout their lifecycle, maintaining the integrity and organization of the sample inventory.

### Remove samples

**Figure 7:**
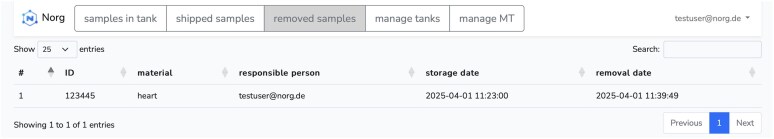
**Remove samples:** It lists all samples that have been removed from the system with detailed information of the responsible person, when the sample was stored and when it was removed.

The removed samples tab lists all samples that have ever been entered into the system but have been removed from it for various reasons (Fig. 7). Here, both the date of storage and the date of deletion are recorded, as well as the responsible person.

### Manage storage

**Figure 8:**
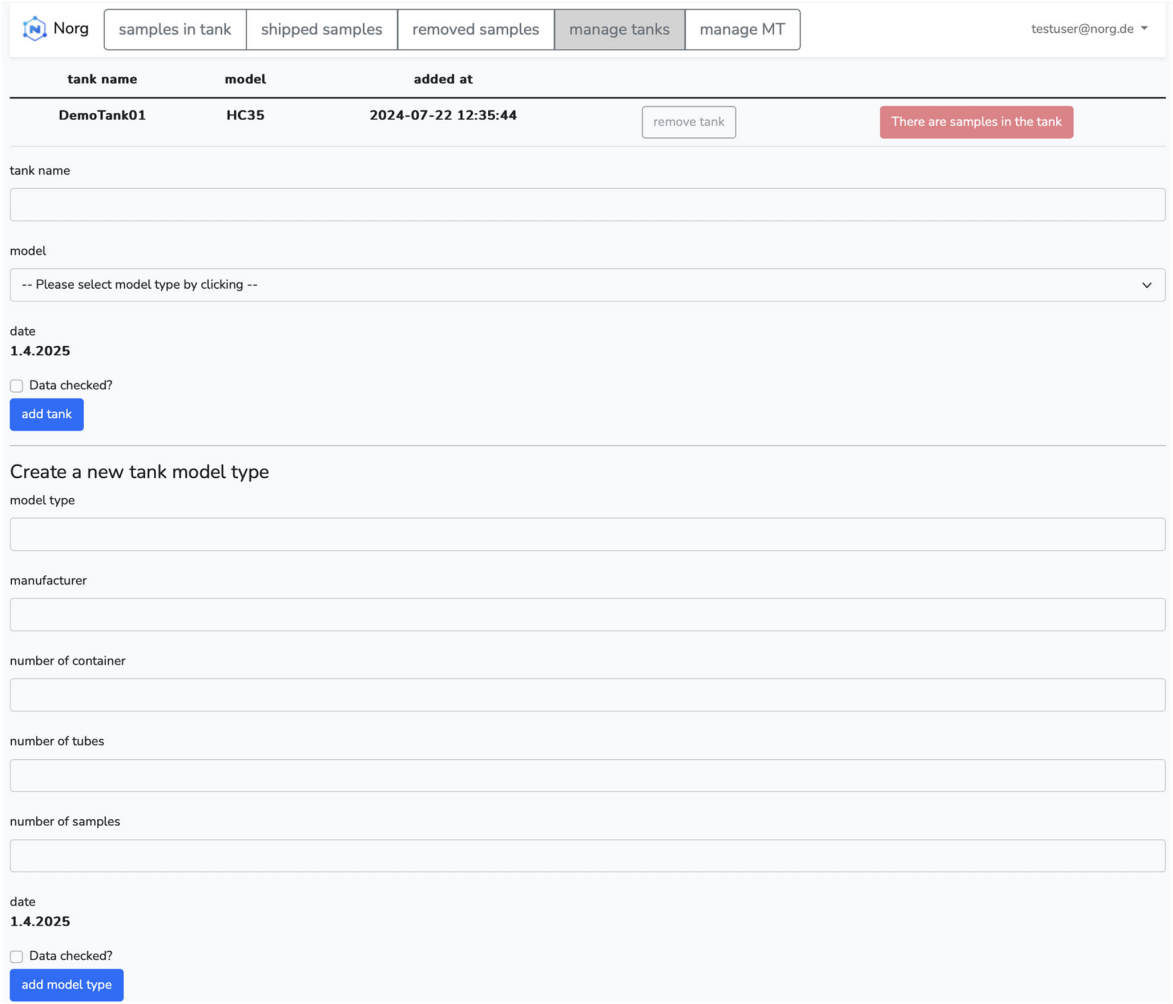
**Manage storages:** This page is designed to manage storages that are in use. One can create a pattern for a certain storage model that can be used later for easily setting up new storages that are used in the laboratory. The storages can be deleted, in case they are empty.

Samples are stored in tanks or freezers that have a defined amount of space. With NORG it is possible to create new storages and provide information about the number of containers, tubes and samples that can be held by the storage (Fig. 8 lower part). In further steps, this storage pattern can be chosen via a dropdown menu when setting up a new empty storage (Fig. 8 upper part). At the top of the page, the existing storages are listed. Here it is also possible to remove storages that were accidentally created or are not used anymore, e.g. because they were discarded. Only containers that do not contain any active samples can be removed.

### Manage users

**Figure 9:**
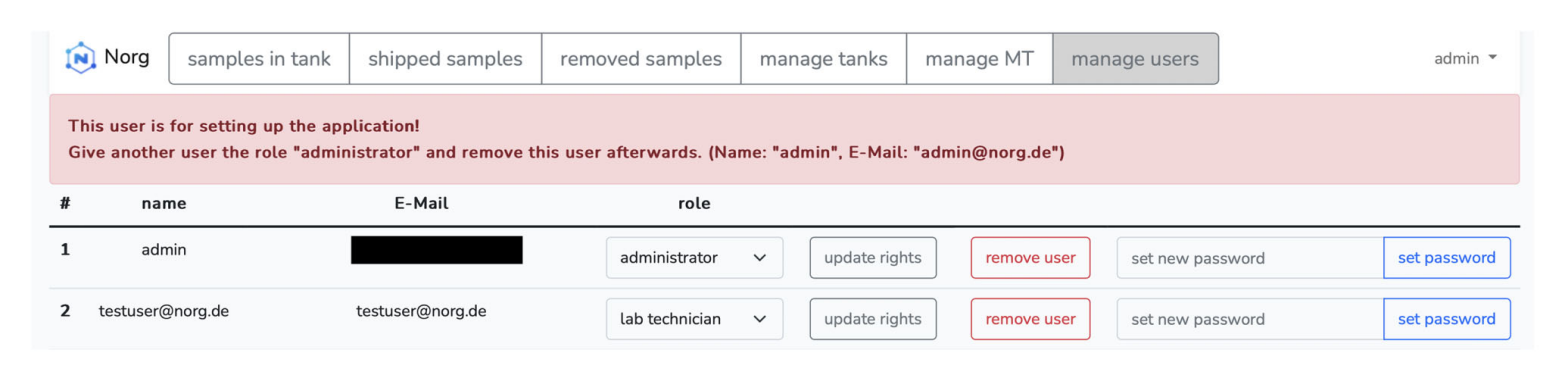
**Manage users:** The user management is a key feature of the administrator. Here, newly registered users are shown and can be accepted. Passwords can be changed by the administrator and users can be removed from the system.

The section for managing users is reserved for administrators (Fig. 9). There is the possibility, as described above, to change the roles of the users. You can see the names, as well as the deposited email addresses, as well as assign the prefabricated roles over the dropdown menu, which are taken over with a click on ‘Update rights’. It is possible to reset forgotten passwords.

### Manage material

**Figure 10:**
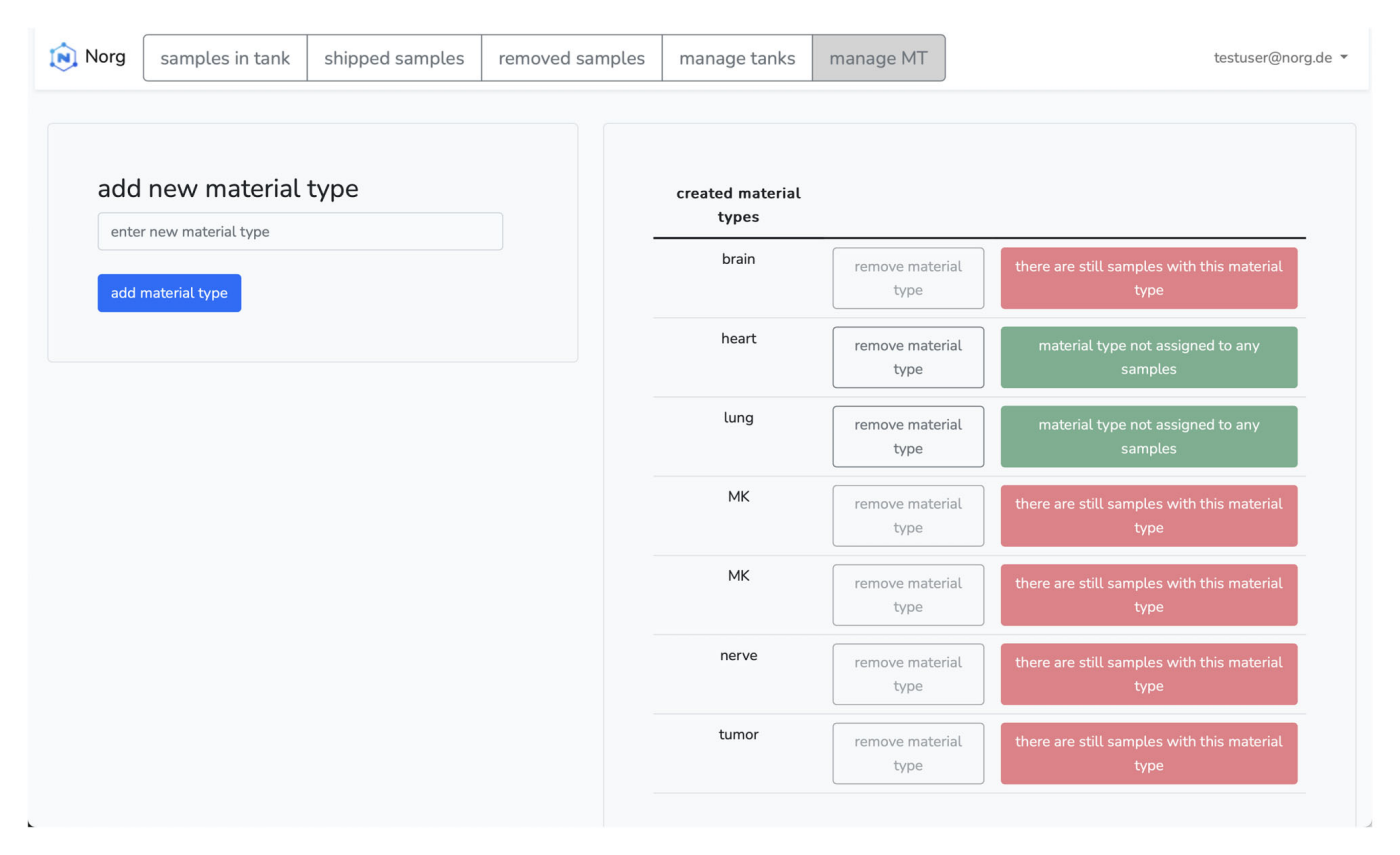
**Manage material:** The users may add new material types to the database, simply by typing their names. A material can only be removed, if necessary, when no sample of that type is stored anymore.

The type of material, used in the different laboratories may strongly vary. We therefore developed a very convenient solution, to manage the material types (Fig. 10). The users may enter a new material on the left, by typing the name of the material or the abbreviation. Already created material can only be removed from the database, when no sample with the particular material is left in the database.

### Import samples from CSV

Transitioning to the NORG system from a different platform, especially when sample data is stored in a CSV file, is streamlined through NORG’s web application. The following steps outline the procedure for importing existing sample data into NORG:

First, it is crucial to ensure that your CSV file is correctly formatted according to NORG’s specifications. Each entry in the CSV file must contain essential information for a single sample, including a storage location (comprising tank name, position insert, position tube, and position sample), a sample identifier, a type of material, a responsible person, and a storage date. Once your CSV file is properly prepared, log in to the NORG web application using your credentials. Before initiating the import process, certain preparatory steps within the NORG system are necessary to match the information in your CSV file. Specifically, you need to verify that the responsible persons, material types, and storage tanks referenced in your CSV file are already present in NORG. If any of these elements are missing, they must be added to the system beforehand. After these preparations, navigate to the import section within the NORG dashboard and select the ‘CSV Import’ option. Upload your prepared CSV file through this interface. The import process begins with a file validation step, ensuring the uploaded file meets size and format requirements. If the file is missing or empty, an error message is generated. The CSV file is then read and processed, with empty rows removed to ensure data integrity. The file is further validated to ensure it contains the expected number of columns and valid header names. During data processing, each row is checked for completeness and accuracy. The system verifies the presence of necessary identifiers and checks if the material types and responsible persons exist in the NORG database. If any required information is missing or if there are discrepancies, the import process is halted, and appropriate error messages are provided. For valid data entries, the import process proceeds to insert the data into the database. If any duplicates are detected—either in the CSV file itself or in comparison with existing database records—an error message detailing the duplicates is generated. The system identifies duplicates based on a combination of storage location attributes and sample identifiers. Upon completion of the import process, NORG will notify you within the system, indicating whether the import was successful. This notification will help you verify that all sample data has been accurately integrated into the NORG platform, ensuring a seamless transition from your previous system.

### FHIR integration

**Figure 11:**
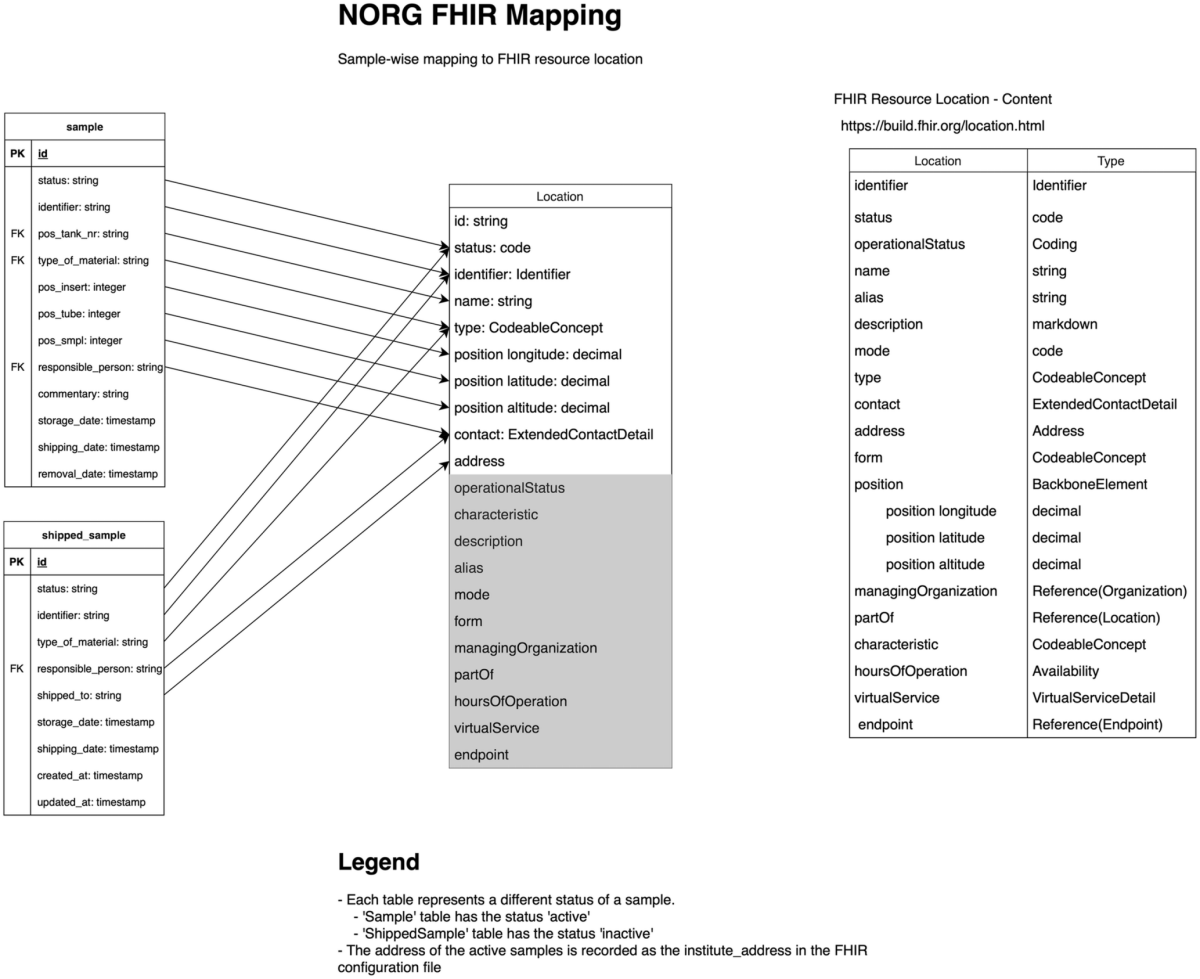
**FHIR mapping:** This diagram illustrates the integration of FHIR resources within the NORG System. The location resource is used to represent physical storage locations and their attributes, such as operational status, managing organization, and virtual service endpoints. Users can manage samples, including creation, shipping, re-storage, and deletion, while ensuring accurate and flexible data sharing across healthcare systems.

FHIR (Fast Healthcare Interoperability Resources) capabilities are integrated into the NORG System, improving the management and sharing of biological samples (Fig. 11). Making it easier to share data across healthcare systems. Using the FHIR Location resource is an important part of NORG’s FHIR integration. This resource is used to represent the physical locations where samples are stored, as well as the sample’s status, the identifiers, the institution’s address and further information.

The system enables comprehensive sample management, including creation, shipping, re-storage, and deletion, all within the FHIR framework. Users can flexibly manage and share data by enabling or disabling FHIR capabilities within the *config/fhir.php* file according to their needs. To ensure correct representation within the FHIR ecosystem, users can set their institution’s address in the *config/fhir.php* file. The NORG System setup includes a Docker Compose configuration that creates an FHIR server, resulting in a ready-to-use environment for FHIR-related tasks.

## Concluding remarks

The here presented NORG system is designed to work as a stand-alone solution without large overheads of additional software, like a laboratory information system. It is intended to keep track of conserved samples with the simplicity of common spreadsheet tools, but with a minimum of user role management. We therefore provided the source code and installation manual at GitHub: https://github.com/Neuropathology-Giessen/NORG. The system is dockerized and works with docker-compose for convenient usage on Linux OS server systems and can be accessed via a standard web browser.

## Data Availability

The NORG database is intended to be used locally for internal tracing of samples. Therefore, a public database is not target-oriented. Nevertheless, we provide a test server system at https://gin-norg-dev.med.uni-giessen.de/ with the credentials *testuser@norg.de* and *testpassword*.
